# Conformal LATP surface engineering for Ni-rich cathodes: enhancing interfacial stability and thermal safety in lithium-ion batteries

**DOI:** 10.3389/fchem.2025.1708033

**Published:** 2025-10-10

**Authors:** Yunli Xu, Lan Wang, Jie Geng, Lin Ma, Jia Qiu, Gaige Han

**Affiliations:** 1 Zhejiang Institute of Quality Science, and Zhejiang Key Laboratory of Consumer Product Safety Research Under Provincial Market Supervision, Hangzhou, China; 2 China Research Institute of Regulation and Public Policy, Zhejiang University of Finance and Economics, Hangzhou, China; 3 Tianfeng Power Supply Co., Ltd., Hangzhou, China

**Keywords:** Ni-rich layered oxide cathodes, LATP coating, interfacial stability, thermal runaway, lithium-ion batteries

## Abstract

Enhancing the interfacial stability and thermal safety of Ni-rich layered oxide cathodes remains a critical challenge for the development of high-energy lithium-ion batteries. Herein, a conformal NASICON-type Li_1_._3_Al_0_._3_Ti_1_._7_(PO_4_)_3_ (LATP) coating was applied to the surface of NCM811 particles via a facile wet-chemical method followed by thermal treatment. Transmission electron microscopy and energy-dispersive X-ray spectroscopy confirmed the uniform distribution of an amorphous LATP layer (∼5–10 nm) on the cathode surface without penetrating the bulk. This LATP coating effectively suppressed interfacial side reactions, stabilized the electrode–electrolyte interface, and mitigated transition metal dissolution, resulting in significantly improved cycling stability and lower impedance growth during electrochemical operation. Importantly, comprehensive thermal runaway evaluations using pouch cells revealed that LATP modification increased the onset (T_1_) and trigger (T_2_) temperatures, extended the delay time to thermal runaway (Δt_1_), and reduced the maximum temperature (T_3_) and mass loss during abuse conditions. These improvements were preserved even after cycling-induced degradation (75% state of health), underscoring the coating’s robustness. This study demonstrates a viable surface engineering strategy that synergistically enhances the electrochemical performance and intrinsic thermal safety of Ni-rich cathodes, providing valuable insights for the design of next-generation safe, high-energy lithium-ion batteries.

## Introduction

With the rapid development of electric vehicles and large-scale energy storage, the demand for advanced battery systems with higher energy density, longer cycle life, and enhanced safety performance continues to rise. Among various battery technologies, lithium-ion batteries (LIBs) have emerged as the most competitive candidates owing to their high energy efficiency and mature manufacturing foundation (Jeevan et al., 2021; Khan et al., 2023; Ming-Wu et al., 2025). Among various cathode materials, LiNi_0_._8_Co_0_._1_Mn_0_._1_O_2_ (NCM811) has attracted considerable interest due to its high nickel content, which enables a high reversible capacity (>200 mAh g^−1^) and energy density while reducing reliance on expensive and toxic cobalt. Furthermore, the layered α-NaFeO_2_-type structure of NCM811 provides favorable lithium diffusion pathways and electrochemical activity (Luo et al., 2022; Qing et al., 2022). Despite these advantages, NCM811 suffers from a series of critical issues that hinder its widespread application. One of the major concerns is its poor thermal stability, particularly at high states of charge (SOC), where structural degradation, oxygen release, and exothermic reactions can be triggered (Chu et al., 2024; Wei et al., 2025; Yuan et al., 2024). These issues are exacerbated under abuse conditions such as overcharge, external heating, or internal short circuits, significantly increasing the risk of thermal runaway (Jin et al., 2021; Lin et al., 2025). Additionally, the high Ni content makes the material more prone to surface phase transitions (e.g., from layered to rock-salt phases), gas evolution, and side reactions with liquid electrolytes, which collectively deteriorate both cycling performance and safety (Cui et al., 2023; Cui et al., 2025; Wang et al., 2025; Wu et al., 2024).

To address the structural and thermal instability of Ni-rich cathode materials such as NCM811, a wide range of strategies have been proposed, including elemental doping, crystal structure modulation, electrolyte optimization, and, most prominently, surface coating (Dianwei et al., 2022; Moin et al., 2024; Wang et al., 2022). Among these, surface modification has shown particular promise due to its ability to directly regulate interfacial reactions between the cathode and the electrolyte, which are often the origin of thermal degradation and gas evolution. By depositing a thin, chemically stable layer on the particle surface, coatings can inhibit the formation of unstable surface phases (e.g., rock-salt NiO), suppress electrolyte decomposition, and limit the dissolution of transition metals (Bandpey et al., 2023). Furthermore, such coatings can serve as physical barriers to moisture and HF attack during storage and cycling, thus improving both shelf life and electrochemical durability. A variety of materials have been investigated for surface coatings, including inert oxides (e.g., Al_2_O_3_, ZrO_2_), conductive oxides (e.g., LiNbO_3_, Li_2_ZrO_3_), phosphates (e.g., Li_3_PO_4_, AlPO_4_), fluorides (e.g., LiF, AlF_3_), and solid-state electrolytes (e.g., Li_7_La_3_Zr_2_O_12_, Li_1_._4_Al_0_._4_Ti_1_._6_(PO_4_)_3_) (Sachs et al., 2015). Some of these materials offer high thermal resistance and chemical inertness, while others provide enhanced lithium-ion conductivity at the interface. Despite these advancements, several challenges persist. Inert coatings can hinder lithium transport, leading to increased interfacial resistance and poor rate performance. Ion-conductive coatings, while beneficial for kinetics, may suffer from poor compatibility with Ni-rich surfaces or undergo degradation during extended cycling at high voltages. Moreover, many coating processes involve complex or high-temperature treatments that are difficult to scale industrially or may alter the bulk structure of NCM materials (Keshavarz et al., 2024; Sun et al., 2023; Wang et al., 2023). More critically, most studies have focused on electrochemical improvements, while their thermal behavior under abuse conditions such as exothermic onset temperature, heat generation rate, and safety performance in practical full-cell formats has not been systematically evaluated. As a result, the question of how to design a surface modification that not only enhances electrochemical stability but also provides substantial mitigation of thermal hazards remains largely unresolved. This limitation underscores the urgent need for multifunctional coating strategies that combine interfacial protection, ionic conductivity, thermal stability, and practical scalability.

In this work, a novel surface modification strategy was developed by uniformly coating lithium titanium aluminum phosphate (Li_1_._3_Al_0_._3_Ti_1_._7_(PO_4_)_3_, abbreviated as LATP) onto NCM811 particles (denoted as NCM-LATP). LATP is a NASICON-type solid electrolyte known for its high lithium-ion conductivity, wide electrochemical stability window, and outstanding thermal robustness, making it a promising interfacial material for enhancing both safety and performance of high-Ni cathodes. The NCM-LATP composite was synthesized via a scalable wet-chemical route followed by thermal treatment, and was subsequently used to assemble pouch-type lithium-ion cells. The thermal stability of the modified cells was systematically evaluated under simulated abuse conditions. Notably, the LATP-coated cells exhibited significantly improved thermal characteristics compared to uncoated NCM811. The onset temperatures of gas release and thermal runaway (T1 and T2) were both elevated, indicating that the modified system is more resistant to triggering thermal reactions under ambient temperature and fully charged conditions. This enhancement suggests that the LATP layer effectively suppresses initial oxygen evolution and interfacial side reactions. Moreover, the maximum temperature during thermal runaway (T3) was reduced, implying that even if thermal runaway occurs, the reaction intensity and associated heat generation are mitigated to some extent. These findings demonstrate that LATP coating not only delays the onset of thermal runaway but also reduces its severity, offering a dual benefit in thermal safety. By integrating the multifunctional properties of LATP into the cathode surface, this study provides a practical and scalable approach toward safer high-energy-density lithium-ion batteries and contributes valuable insights into the rational design of thermally stabilized Ni-rich cathode materials.

## Experimental section

### Materials synthesis

. Commercial LiNi_0_._8_Co_0_._1_Mn_0_._1_O_2_ (NCM811, Beijing Easpring Material Technology Co., Ltd.) was used as the cathode active material. LATP (Li_1_._3_Al_0_._3_Ti_1_._7_(PO_4_)_3_) was synthesized by a sol–gel method: stoichiometric amounts of LiNO_3_, Al(NO_3_)_3_·9H_2_O, Ti(OC_4_H_9_)_4_, and NH_4_H_2_PO_4_ were dissolved in ethanol and deionized water, followed by continuous stirring to form a homogeneous solution. The obtained sol was dried at 80 °C and subsequently calcined at 750 °C for 6 h in air to obtain crystalline LATP powder. The coating process was conducted by dispersing LATP precursor in ethanol and uniformly mixing with NCM811 powder, followed by drying h, resulting in NCM811-LATP composites.

### Characterization

The morphology and microstructure were observed using scanning electron microscopy (SEM, Hitachi S-4800, 5 kV) and transmission electron microscopy (TEM, JEOL JEM-2100F, 200 kV). The crystal structure was analyzed by X-ray diffraction (XRD, Bruker D8 Advance, Cu Kα radiation, λ = 1.5406 Å) in the 2θ range of 10°–80°.

### Cell assembly

Cathodes were prepared by mixing NCM811 or NCM811-LATP, Super P, and PVDF in N-methyl-2-pyrrolidone (NMP) at a mass ratio of 8:1:1. The slurry was coated onto aluminum foil, dried under vacuum at 120 °C for 12 h, and then roll-pressed. Pouch-type half cells were assembled in an Ar-filled glovebox, using sodium metal as counter/reference electrode, glass fiber (Whatman GF/D) as separator, and 1 M NaPF_6_ in diglyme as electrolyte.

### Electrochemical testing

Galvanostatic charge–discharge cycling was carried out on a LAND CT2001A battery test system. Cyclic voltammetry (CV) and electrochemical impedance spectroscopy (EIS) were conducted on a CHI660E electrochemical.

## Results and discussions

**FIGURE 1:**
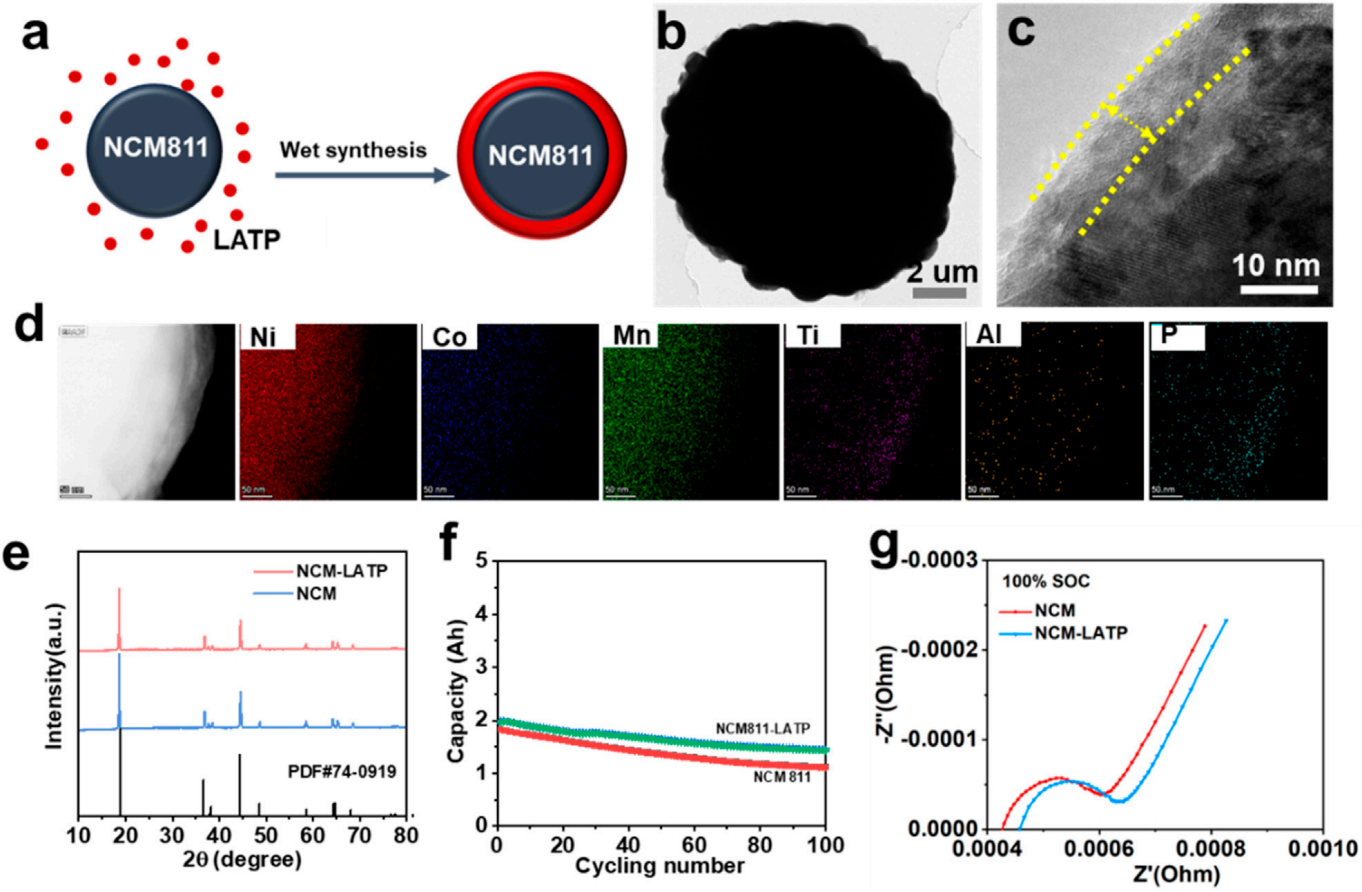
**(a)** Schematic illustration of the LATP surface modification strategy. **(b)** TEM image of pristine NCM-LATP. **(c)** High resolution TEM image of NCM811-LATP **(d)** EDS elemental mapping of NCM811-LATP. **(e)** XRD patterns of NCM-LATP and NCM. **(f)** Cycling performance at 0.5C and 25 °C. **(g)** Electrochemical impedance spectra at 100% state of charge.

To improve the interfacial stability and thermal robustness of Ni-rich layered cathodes, a LATP coating was applied to NCM811 particles via a mechanical fusion method followed by thermal annealing. The synthesis route is schematically illustrated in Figure 1a. LATP precursors were homogeneously deposited onto the surface of NCM811 particles and subsequently crystallized to form a conformal coating layer. This surface engineering strategy aims to integrate the high ionic conductivity and thermal stability of NASICON-type LATP with the electrochemical performance of high-energy cathode materials. The morphology and microstructure of the coated material were characterized using transmission electron microscopy (TEM). As shown in Figure 1b, the pristine NCM811 particle exhibits a dense and well-defined morphology. Upon LATP coating, a distinct and uniform amorphous layer with a thickness of approximately 5–10 nm is clearly observed at the particle surface (Figure 1c), indicating the successful formation of the LATP layer. This conformal coating is expected to act as a physical barrier that suppresses direct contact between the cathode surface and the electrolyte, thereby reducing interfacial side reactions and mitigating parasitic heat release during thermal abuse. To further verify the elemental distribution, energy-dispersive X-ray spectroscopy (EDS) mapping was conducted, as shown in Figure 1d. The Ni, Co, and Mn signals are uniformly distributed throughout the particle, consistent with the NCM811 structure. Importantly, the elements Ti, Al, and P—characteristic of LATP—are clearly detected and predominantly located at the particle periphery, confirming the successful deposition of LATP on the surface without significant penetration into the bulk structure. The well-defined spatial separation of the coating and core regions supports the structural integrity of the NCM811 host material. The crystallinity of the amorphous LATP was examined by X-ray diffraction (XRD). As shown in the XRD patterns (Figure 1e), the pristine NCM811 cathode matches well with the standard card (JCPDS No. 74-0919), confirming the preserved layered structure. After LATP coating, the XRD pattern of NCM811/LATP remains unchanged without any new diffraction peaks corresponding to crystalline LATP, which indicates that the LATP layer is amorphous. The impact of LATP surface modification on electrochemical performance was evaluated by galvanostatic cycling at 0.5 °C and 25 °C. As depicted in Figure 1f, the NCM811-LATP electrode exhibits improved capacity retention over 100 cycles compared to pristine NCM811. The enhanced cycling stability is attributed to the LATP layer’s ability to suppress surface degradation, minimize transition metal dissolution, and stabilize the cathode–electrolyte interface. The LATP-coated electrode retains ∼75% of its initial capacity after 100 cycles, whereas the pristine sample shows a more pronounced capacity fade. These results suggest that the coating effectively preserves structural and electrochemical integrity during repeated lithium extraction/insertion. Electrochemical impedance spectroscopy (EIS) further reveals the beneficial effect of LATP modification. As shown in Figure 1g, the NCM811-LATP cell exhibits a smaller semicircle in the high-to-medium frequency range at 100% state-of-charge (SOC), indicating lower interfacial resistance compared to uncoated NCM811. This suggests that the LATP layer facilitates Li^+^ transport across the electrode–electrolyte interface and suppresses the formation of resistive interphase layers during cycling. Despite the presence of a coating, no significant increase in impedance is observed, confirming that the LATP layer maintains good ionic conductivity while enhancing surface stability.

**FIGURE 2:**
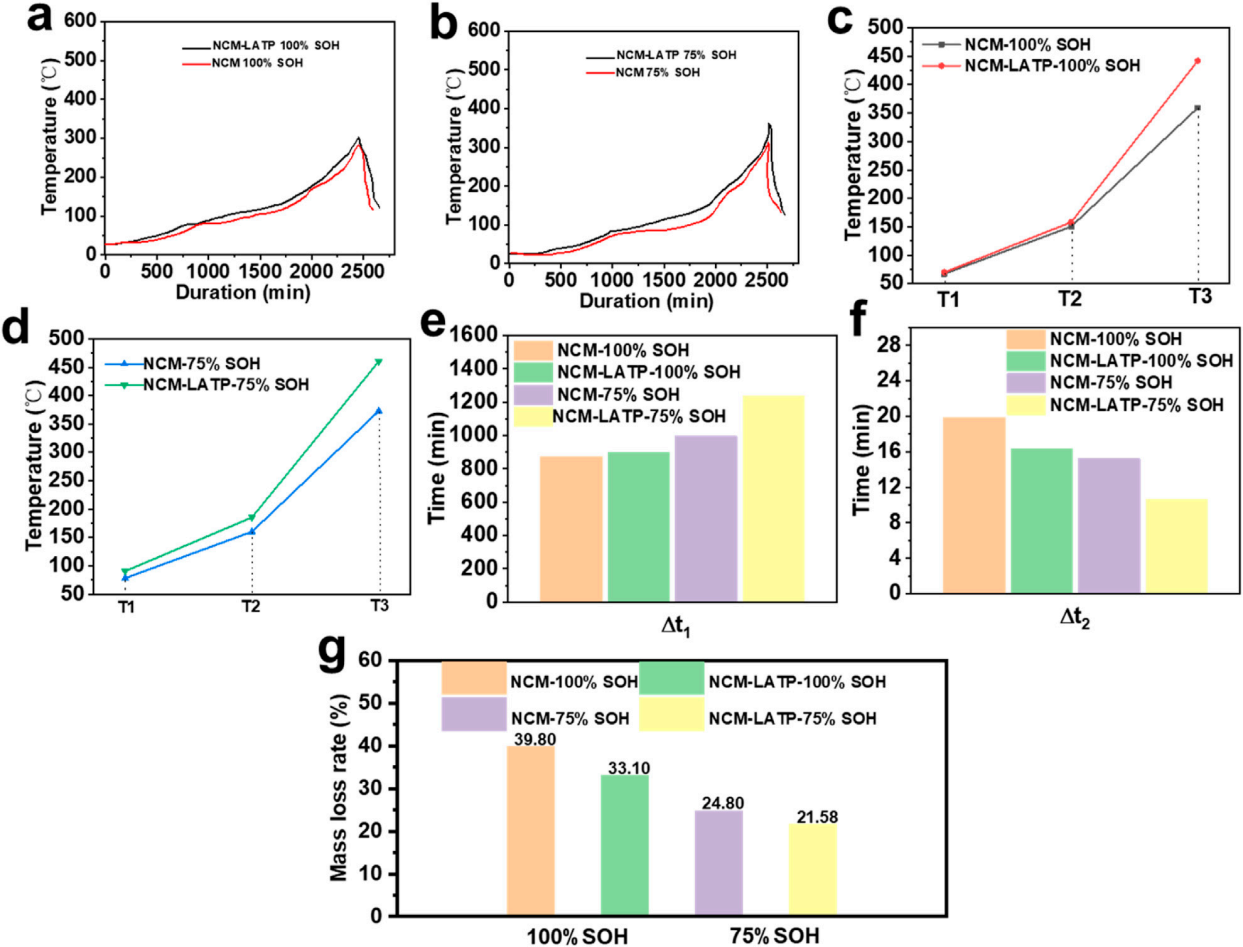
Thermal runaway behavior of pristine and LATP-coated NCM811 pouch cells at different states of health. **(a)** 100% SOH. **(b)** 75% SOH. **(c)** Thermal runaway temperature profiles (T_1_, T_2_, T_3_) of pristine and LATP-coated cells at 100% SOH. **(d)** Thermal runaway temperature profiles (T_1_, T_2_, T_3_) of pristine and LATP-coated cells at 75% SOH. **(e)** Δt_1_ under different SOH conditions. **(f)** Δt_2_ for pristine and LATP-coated cells. **(g)** Mass loss rate after thermal runaway events.

To evaluate the thermal stability and safety benefits imparted by the LATP surface coating, a series of thermal abuse tests were conducted on both pristine and LATP-modified NCM811 soft-pack cells under different states of health (SOH), including 100% SOH and 75% SOH conditions (Figures 2a,b). The evolution of characteristic thermal runaway indicators—including onset temperature (T_1_), thermal runaway triggering temperature (T_2_), peak temperature (T_3_), delay time to thermal runaway (Δt_1_), reaction time (Δt_2_), and mass loss rate—was systematically compared, as summarized in Figures 2c–g. Under 100% SOH conditions (Figure 2c), the LATP-coated NCM811 cell exhibits a delayed onset of exothermic activity, with the initial temperature rise (T_1_) occurring at 70 °C, notably higher than that of the pristine NCM811 counterpart. The triggering temperature for thermal runaway (T_2_) increases from 158 °C in pristine NCM811 to 164 °C in the LATP-coated sample, indicating that the coating effectively elevates the thermal threshold required for initiating self-accelerating reactions. In contrast to the suppressed early-stage reactions, the peak temperature (T_3_) of the LATP-coated sample reaches 352 °C, which is higher than that of pristine NCM811 (342 °C), suggesting that more heat accumulates before the system reaches its maximum thermal release, possibly due to the delayed but more concentrated heat evolution. At 75% SOH (Figure 2d), where battery degradation typically exacerbates thermal instability, the LATP-coated electrode continues to exhibit enhanced thermal resilience. The T_1_ and T_2_ values further increase to 82 °C and 164 °C, respectively, compared to 78 °C and 158 °C in the degraded pristine cells. Notably, the T_3_ value for NCM811–LATP rises to 362 °C, again higher than that of uncoated NCM811, indicating that although the onset and trigger of thermal runaway are significantly delayed, the eventual thermal release remains substantial, albeit more controlled. The elevated onset and trigger temperatures observed at both SOH states confirm that the LATP layer serves as an effective thermal barrier, postponing the initiation of interfacial decomposition reactions at the cathode–electrolyte interface.The delay time to thermal runaway (Δt_1_), defined as the duration from external heating to the occurrence of uncontrollable self-heating, is presented in Figure 2e. At 100% SOH, the pristine cell undergoes thermal runaway after 874 min, whereas the LATP-coated cell resists thermal runaway for a prolonged period of 897 min. The protective effect becomes more pronounced under 75% SOH, where Δt_1_ increases from 996 min (pristine) to 1238 min (LATP-coated), representing a substantial enhancement of over 24%. This extended delay window offers a critical advantage for early detection, active intervention, and failure mitigation in practical applications. The reaction time (Δt_2_), defined as the interval between the thermal runaway trigger and peak temperature, reflects the reaction kinetics during thermal runaway. As shown in Figure 2f, LATP coating significantly accelerates the heat release process once runaway is initiated, with Δt_2_ values of 16 and 10 min for 100% and 75% SOH, respectively, compared to 20 and 15 min for pristine samples. The shortened Δt_2_ is indicative of a more moderated and less violent runaway process, likely due to the absence of uncontrolled interfacial reactions and suppressed oxygen evolution from the cathode lattice. Rather than prolonging the exothermic phase, the LATP layer promotes a faster energy release in a more manageable and less catastrophic fashion.The mass loss rate, as determined by post-mortem analysis (Figure 2g), provides further insight into the extent of material degradation during thermal runaway. At 100% SOH, pristine NCM811 cells exhibit a mass loss of 39.8%, whereas the LATP-coated cells show a reduced value of 33.0%. This trend is even more apparent at 75% SOH, where mass loss decreases from 24.8% (pristine) to 21.58% (LATP-coated). The protective role of the amorphous LATP coating can be summarized as follows. The phosphate-rich, amorphous LATP layer is ionically conductive but electronically insulating, forming a stable interphase with the Ni-rich surface. (i) The coating suppresses early electrolyte oxidation and lattice oxygen release, thereby increasing the onset temperature (T1). (ii) By reducing active sites for parasitic reactions and moderating heat generation kinetics, the delay time to thermal runaway (Δt_1_) is prolonged. (iii) The coating mitigates hot-spot formation and delays the coupling of exothermic reactions with separator failure, leading to a higher trigger temperature (T2). (iv) In addition, the LATP layer restrains electrolyte penetration and volatile formation, which lowers the maximum temperature (T3) and mass loss during runaway. The amorphous structure also enhances interfacial conformity and accommodates stress, maintaining integrity under abuse. Together, these effects account for the observed improvements in thermal stability. The mechanistic explanations of how the amorphous LATP layer improves thermal stability have been added to the revised manuscript. Taken together, the comprehensive thermal analysis confirms that LATP surface modification significantly enhances the thermal safety of Ni-rich layered cathodes. By elevating the onset and trigger temperatures, extending the thermal delay window, reducing material degradation, and moderating the runaway dynamics, the LATP coating effectively mitigates the severity and probability of thermal runaway events. This multifaceted improvement underscores the critical importance of interfacial engineering in enabling high-energy-density lithium-ion batteries with enhanced operational safety.

## Conclusion

In summary, a conformal NASICON-type LATP coating was successfully constructed on the surface of Ni-rich NCM811 cathode materials via a simple wet-chemical deposition followed by thermal annealing. Structural and compositional analyses confirmed the uniform distribution of LATP at the particle surface without altering the bulk integrity. This rational surface engineering strategy significantly enhanced the interfacial stability between the cathode and the electrolyte, leading to improved capacity retention and reduced interfacial impedance during cycling. More importantly, systematic thermal abuse tests revealed that the LATP coating effectively elevated the onset and trigger temperatures of thermal runaway, extended the delay time before catastrophic events, and reduced both the exothermic intensity and material loss under both fresh and aged conditions. These improvements are attributed to the high thermal stability, ionic conductivity, and chemical inertness of the LATP layer, which acts as a multifunctional barrier to suppress interfacial side reactions and mitigate thermal propagation. This work highlights the critical role of interfacial regulation in simultaneously achieving high electrochemical performance and intrinsic safety in next-generation high-energy lithium-ion batteries.

## Data Availability

The original contributions presented in the study are included in the article/supplementary material, further inquiries can be directed to the corresponding author.
