# Correction: Conformal LATP surface engineering for Ni-rich cathodes: enhancing interfacial stability and thermal safety in lithium-ion batteries

**DOI:** 10.3389/fchem.2025.1724562

**Published:** 2025-11-03

**Authors:** Yunli Xu, Lan Wang, Jie Geng, Lin Ma, Jia Qiu, Gaige Han

**Affiliations:** 1 Zhejiang Institute of Quality Science, and Zhejiang Key Laboratory of Consumer Product Safety Research Under Provincial Market Supervision, Hangzhou, China; 2 China Research Institute of Regulation and Public Policy, Zhejiang University of Finance and Economics, Hangzhou, China; 3 Tianfeng Power Supply Co., Ltd., Hangzhou, China

**Keywords:** Ni-rich layered oxide cathodes, LATP coating, interfacial stability, thermal runaway, lithium-ion batteries

There was a mistake in Figure 1 as published. The panels labeled (a) and (f) did not correspond to the actual experimental results because the wrong file was selected during submission. The corrected figure appears below.

**FIGURE 1:**
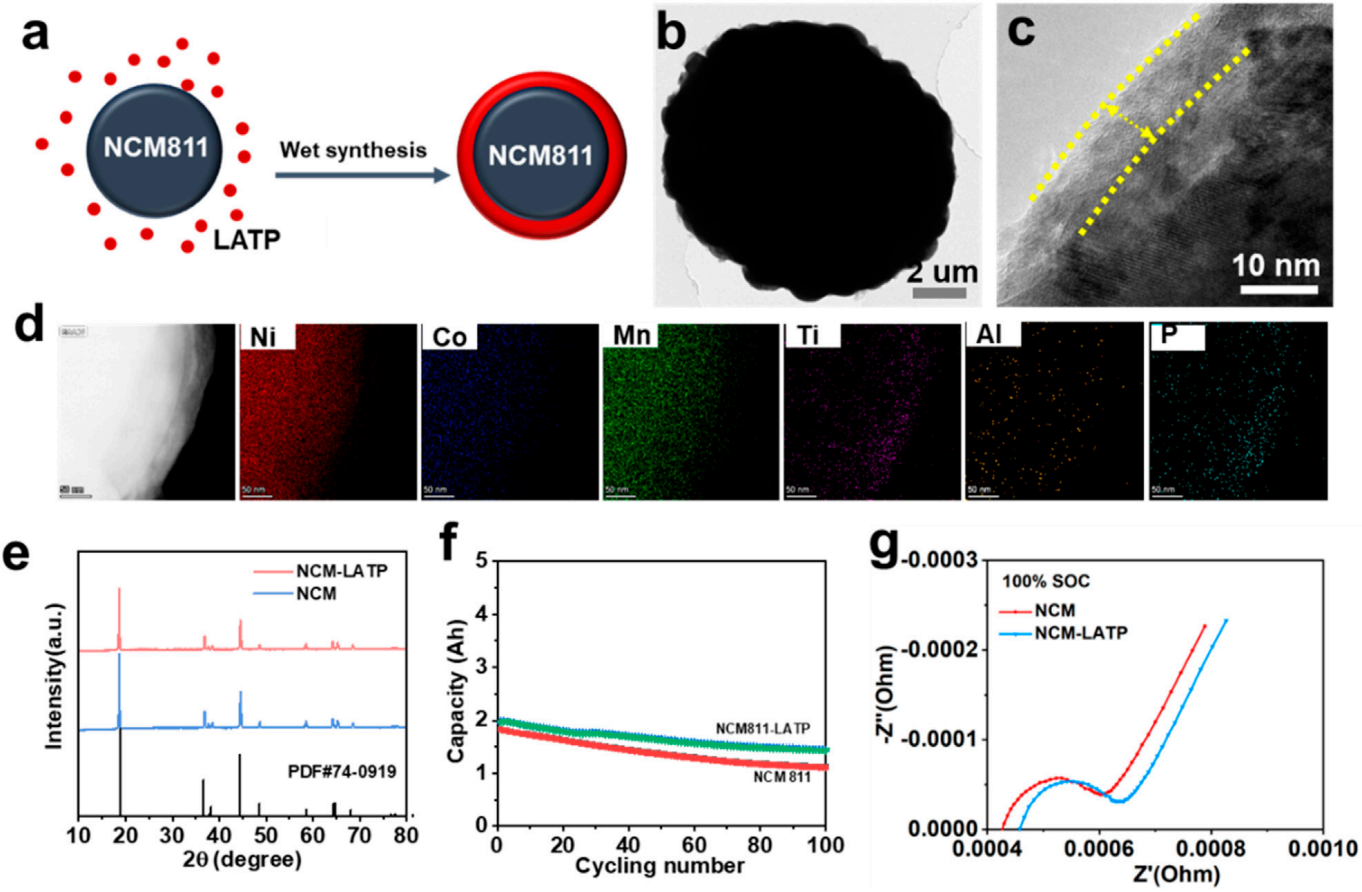
**(a)** Schematic illustration of the LATP surface modification strategy. **(b)** TEM image of pristine NCM-LATP. **(c)** High resolution TEM image of NCM811-LATP **(d)** EDS elemental mapping of NCM811-LATP. **(e)** XRD patterns of NCM-LATP and NCM. **(f)** Cycling performance at 0.5C and 25 °C. **(g)** Electrochemical impedance spectra at 100% state of charge.

The original article has been updated

